# The emotional schemas of psychiatric patients- a case-control study

**DOI:** 10.1192/j.eurpsy.2021.1251

**Published:** 2021-08-13

**Authors:** I. Rivis, I. Papavă, M. Minciună, A. Bredicean, S. Ursoniu

**Affiliations:** 1 Neurosciences, “Carol Davila” University of Medicine and Pharmacy, Bucharest, Romania; 2 Neurosciences, “Victor Babes” University of Medicine and Pharmacy, Timisoara, Romania; 3 Psychiatry, “Pius Brânzeu” Emergency County Hospital, Timisoara, Romania; 4 Functional Sciences, “Victor Babes” University of Medicine and Pharmacy, Timisoara, Romania

**Keywords:** emotional schemas, mental health, LESS, Psychiatric disorders

## Abstract

**Introduction:**

Our Emotional Schemas dictate how we deal with our own emotions, therefore, how we interpret and face different events that occur in our everyday life. Maladaptive schemas have been proven to be at fault for the inability to face different challenges.

**Objectives:**

This study aims to find the differences in emotional schemas between subjects with history of psychiatric disorder and subjects without a psychiatric disorder.

**Methods:**

We realized a case-control study matched for age and gender, and analyzed the answers of 28 subjects (14 women and 14 men) to Leahy Emotional Schema Scale (LESS); 14 of which have a personal history of psychiatric disorders, while the remaining 14 had no such history. The LESS evaluation was part of a bigger study and was addressed to the general population, over 18 years old. The test was applied online, with the informed consent of the subjects.

**Results:**

The mean age of the participants was 40.28±13.98. Out of the 14 subjects with a psychiatric diagnosis, 71,43% have a job, 21,43% are retired and 1% are still studying. There was a significant difference between the two groups regarding the Higher Values dimension of the Emotional Schemas (p=0.0419). Also, the question regarding the feeling of shame when it comes to their own feeling, showed significant difference between the two groups (p=0.0211).

**Conclusions:**

As opposed to the subjects without a history of psychiatric disorder, those who do have a psychiatric diagnosis, feel more often devalued and ashamed, therefore having a lower self-esteem.

